# Space-technological and architectural methodology and process towards design of long-term habitats for scientific human missions on mars

**DOI:** 10.1016/j.mex.2023.102270

**Published:** 2023-06-28

**Authors:** Kasra Amini, Mojgan Moradi, Bahareh Vossoughi, Ehsan Dehghani Janabadi

**Affiliations:** a FLOW and Fluid Physic Laboratory, Department of Engineering Mechanics, Royal Institute of Technology (KTH), Stockholm, Sweden; b Department of Architectural Technology, Faculty of Architecture and Urban Planning, University of Art, Tehran, Iran; c Faculty of Mechanical Engineering, RWTH Aachen University, Aachen, Germany

**Keywords:** Martian Habitat Units (MHUS), Human space mission, Modular design, Hierarchical modulation, Closed-loop systems, Martian environment, Exterior flow field control, Iterative Design

## Abstract

Centered on the core idea of long duration habitat design for research crew on Mars, the Martian Habitat Units (MHUs) are designed as a cluster of 10 units each with the maximum capacity of 9 crew members to live and carry on with the local challenges of scientific and exploratory life, while enjoying their lives as intellectual, social individuals in the harsh environment of Mars for durations in the order of magnitude of several years. This approach to the concept of a living environment in sharp contradiction to that of a shelter with the minimal capabilities to meet the requirements of terrestrial life to the point of survival, has led the outcoming design to be a fulfilling environment for the inhabitants of the units to evolve and thrive culturally, while being on a years-long mission. This manuscript provides detailed insight on the *lessons learned* of the aforementioned comprehensive design attempt with, but not limited to, the following core concerns:

• The initial stand-point of such a design procedure relies on an ever increasing and comprehensive list of concerns, be it classically discussed in the literature and predictable, or unforeseen on the face of it, but to be prevented anyhow. The manuscript discusses the most crucial ones of such criteria/concerns.
• The infamous saying of “*Whatever that can go wrong, will go wrong*” demands a rather complex level of redundancies in all layers of the design and the thought procedure behind its all aspects. The manuscript addresses the adequate steps towards its realization.
• Modularity in all layers of the design plays a key role in reducing construction, maintenance, and installation costs, as for any deep space mission the mentioned expenses are astronomically high themselves. The manuscript presents our solution for geometric modularity of the design.

## Graphical abstract

**Figure:**
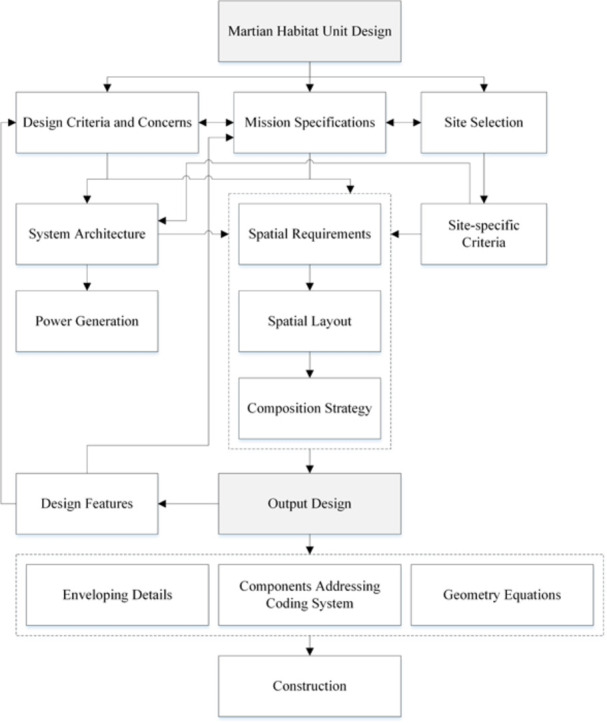

The challenge of designing Martian Habitat Units commences with three main agenda and leads to the design outputs, namely the design criteria and concerns, mission specifications and site selection, the steps of which are summarized in Fig. 1. This will also address the construction concerns as enveloping details, components addressing coding system and the geometric equations.

Specifications table

**Table:** 

Subject area:	Space Technology and Architecture
More specific subject area:	Space Colonization
Method name:	Iterative Design
Name and reference of original method:	Detailed in the linked manuscript to the current Methods article: https://doi.org/10.1016/j.icarus.2022.115119
Resource Availability:	Satellite / Mars Rovers’ Data

## Method details

### Background

Reaching for promising target planets to feed the ever-increasing need of our modern times on the verge of becoming a type-1 civilization, Mars is inevitably the next body to be explored. The quest has already begun and will only accelerate until landing the first astronauts on the planet. This is, of course, in compliance with the need to know more about the conditions on Mars, but deep in itself, it will be an attempt to quench our thirst of knowing that *we can* transfer humans to Mars. Needless to say, that the first to walk on Mars are to seek shelter, live with the minimum they might have been able to land with them on the surface, and spend time with a sort of life, i.e. life conditions, not wished for by many. However, soon after, like all other trends in science and technology, the travel to Mars will become more achievable with lower expenses, and more frequent, as long as the orbital conditions allow, and the current survival-only mission mentality will morph to a more settlement-type inhabitation, at least for the few years duration each astronaut is going to be living on the low gravity, and harsh environment of Mars, before they are sent back to the Earth for retrieval. This is of course the step after the current one we are to witness in the upcoming decades to the problem of human missions to Mars, however, to skip a stem and think ahead for the solutions of the upcoming steps has always been the thrusting engine of science, and so is the scope of the current manuscript. The following table (Table 1) presents a brief glance of the current state of research/technology for long term human habitats.

**Table 1:** State of research – long term human habitat.

Time Period	Paradigm	Feature (s)	Reference Article	Author(s), Year
1990s	Tuna-can shaped habitat	A minimal approach: a two-phased manned Mars mission for exploring and surveying with the prospect of founding a 48-person permanent Mars-base and some colonization thoughts.	Mars Direct: Humans to the red planet by 1999	[1]
	MDRM	A cylinder as an initial design for a total of 8 participants with no pre-positioning launches, dual habitats, pressurized rover, science laboratory or visible airlocks	Human Exploration of Mars: The Reference Mission of the NASA Mars Exploration Study Team	[2]
2000s	The Bologna-slice and Banana-split modules	Two modules designed for 8 people The Bologna-slice: includes circular modules for temporary accommodations, Banana-split: includes a cylindrical module with parallel axes for transportations between the Earth and Mars	Mars habitat modules: Launch, scaling and functional design considerations	[3]
	TransHab	A rigid frame, comprising two conical frustum assemblies at each end (airlocks), 8 fixed axial core components, foldable modular panels and one fixed rigid bottom panel. When deployed, the inflatable structural shell makes a two-story space that contributes to thermal and radiation protection	Variants on the TransHab paradigm 2: The surface endoskeletal inflatable module (SEIM)	[4]
	Hybrid pre-integrated deployable habitats	An inflatable element combined with a “tuna can” module	Planetary base element envelope, layout, and configuration interface considerations	[5]
	FMARS	Two-story cylindrical tuna-can modules made of corrugated fiberglass as insulation and protection against wind, snow, and water damage	Flashline Mars arctic research station (FMARS) 2009 crew perspectives	[6]
2010s	The utilization of native materials (*In Situ* Resources)	The utilization of *In Situ* Resources in assembling habitats, as well as supplying fuel, oxygen and water	The case for Mars: The plan to settle the red planet and why we must	[7]
	Inflatable and dome-shaped modules	Modules shaping the central core, the agricultural part, the residential part, the lab and parking. Each part includes three sections from which, future developments are possible. The residential part is designed as an ''open plan'', which provides options for personalizing the space	Human friendly architectural design for a small Martian base	[8]
	Lava tubes for Martian habitats	An idea of the overall architecture, comprising of two tunnels, as well as the station and transportation system, is suggested. One tunnel is dedicated to housing, privacy and societal functions and the other one to the industrial activities of the station	A roadmap to cave dwelling on the Moon and Mars	[9]
	MDRA 5.0	The emphasis on a science-oriented mission, leading to the design of combined pressurized rovers with hybrid monolithic habitats, in which there is an inflatable module on top. There is no proximity in IPV and habitats as in previous designs	First Mars habitat architecture	[10]
	Use of 3D printers	A special expandable module recommended for the use of 3D printers, which includes two nested domes connected together by junctures. The bigger dome will be used as agricultural space, that is a transparent lightweight structure, and inside there are smaller domes as living rooms	Utilizing *in-Situ* resources and 3D printing structures for a manned Mars mission	[11]
	The Ice House	A multilayer design, including a central part and two protecting layers. The first semi-transparent protective layer is made of ETFE, water and aerogel. The second one is the ice shell composed of aerogel	NASA 3D-printed habitat challenge	[12]
	Use of 3D printers, robots, and indigenous materials	A habitat that is to be built in two phases. In the first step robots choose the site and excavate the 1.5 m deep cavity. The second step is to put the inflatable modules in the site. The material chosen for construction is fused regolith due to protecting features against radiation and extreme temperatures. Use of the modular design and assigning separated duties to robots increase redundancy.	NASA 3D-printed habitat challenge	[28]
	Minimum Functionality Habitat	The main idea of designing conceptual minimal habitats with expandable elements to support the reference missions	Space architecture education for engineers and architects: Designing and planning beyond earth	[13]
	Mars mockup projects	It is composed of three nested parts including a central core, an additional living space, and a dome that protects the first and the second spaces. It contains polyethylene fibers pykrete and ice water with a thickness of 3 m	Systems engineering and design of a Mars Polar Research Base with a human crew	[14]

**Fig. 1:**
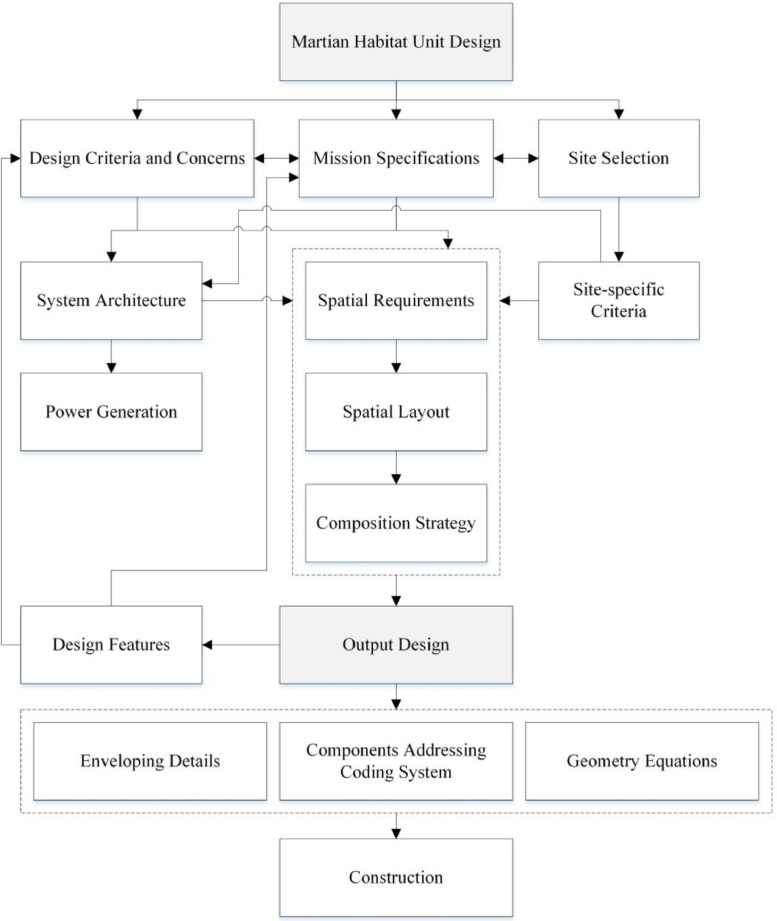
The design process flowchart.

It should be noted that this manuscript parodies a previous paper by the same group of authors [20], where the full detailed discussion on the design and its implications are presented. The current method paper opens with a selected list of concerns shaping the initial direction of the design. Upon detailed definition of the problem for which the designed MHUs are supposed to function, the domain of plausible missions is described, while pointing at the specific components of the base mission defined for the current research. Furthermore, a section on the process of design and its underlying philosophy is followed by the details of the outcoming design; the Martian Habitat Units (MHUs). The geometric specifications, plans and 3D sketches of the design, and features of the case are presented in this section. Next, the power generation systems’ concerns and criteria are discussed followed by insights on the manufacturing and construction planning of the MHUs. The manuscript closes with an illustration on the future works in progress in the full scope of the project and concluding remarks (Fig. 1).

## Design requirements – concerns list

Prior to the project definition, a comprehensive mission specific design requirements and concerns have been gathered based on the standards NASA-STD-3001 vol 1 and 2, DRA 5.0, COSPAR policies on planetary protection and the previous research done in physiological and psychological well-being of the crew in short- and long-term missions [[29], [30], [31], [32]]. The following table (Table 2) is a summary of these categorized concerns and suggested considerations for such a multidisciplinary project.

**Table 2:** Design concerns and considerations.

Category	Design Concerns	Design Considerations
Environmental Hazards	. Low gravity . Radiation . Extreme temperatures . Dust / Dust Storms . Mars quakes . Micrometeorites . Mars rigorous	Eliminating or optimizing EVA / Use of robotics and telepresence (Anderson et al. [15]) / Careful diet and physiology monitoring / Traditional and modern countermeasures such as radioprotectants and pharmaceuticals, advanced shielding and biotechnologies / Artificial gravity / Insulations and coatings / Adjustable external membrane to control the flow field and dust settlement in inner regions of the cluster to protect PVs / Using smart windows and implementing virtual reality / Clean area criteria / Zonal separation / Larger space proportions / Visual aids / High safety factor / Emergency scape paths / Chemical treatment
Habitat Comfort Zone	. Pressurized habitat . Fresh air supply . Water supply . Thermal comfort . Humidity control . Ventilation . Contamination control . Noise control . Waste management . Food and nutrition . Visual comfort . Light . Clothing . Ergonomic habitat	Pressure bearable geometries and materials / Suspended ceilings / Insulations and coatings / Consistent control of the energy loss / Radiators and heat exchangers / Evacuation and quarantine area / Noise insulation and cancelation layers around loud working instruments while providing ease of access for maintenance / Multiple disposal methods such as recycling, furnace and sending back to earth / Considering storage area / Considering the plants grown in the greenhouse as the main source of food / Supplying food from Earth or orbiting stations / Food preparation facility and dining area / Designing a garden area around the residential area and green modules inside the units / Controllable artificial lighting integrated by AI / Light tubes / Considering private and personal hygiene area for each crew member / Using *in-Situ* materials / Omitting the sharp edges in design / Mobility aids
Habitat Spatial Requirements	. Personal hygiene facility . Body waste management . Entrance / Exit . Internal translation paths . Emergency translation paths . Windows . Storage area . Parking . Food preparation . Community area . Personal area . Camp-out area . Garden / Greenspace . Laboratory . Working area . Medical area . Exercise area . Quarantine area . LSS hardware . AI hardware . Telecommunication / Data handling	Zonal separation / Privacy / Water flushers, pumps and spraying water / Suction systems / Waste storage / Ingress and egress loop adjacent to vestibule / Clean room criteria / Separating the work and personal area / Daily schedule / Emergency exit routes and protocols / Windows with outside view / Windows inside the unit to the green areas mainly for psychological reasons / Smart windows / Relative orientation of neighboring units / Preventing the entrance blockage of the parking or the vehicles / Not pressurized parking in normal operation but pressurized when needed for maintenance reasons / Food preparation area close to the community area /Community area equipped with smart windows and large monitors / Personal area equipped with smart windows on second level and separated from loud working systems / Noise control systems / The garden as a buffer zone between inner residential area and outer protection layer, due to possible lower pressure difference and visual comfort considerations / Maintaining a static position and orientation at workstation / Enough lighting / Integrated AI assistance / Hierarchy of access and unilateral gate control / Homogeneous redundancy of LSS / Cyber security measures
Subsystems and Instruments	. LSS . Power generation and storage . Telecommunication . BMS . Robotics . Virtual reality . Expert system . Smart windows . Spacesuits . Lifts . Tool / Equipment / Hardware	High redundancies and large proportion of space to accommodate the physio-chemical LSS hardware / The enveloping garden as the main food source and backup bio-regenerative LSS / AI and robotic aided consistent control of the environment / Emergency escape routes and protocols / Kilopower 10 kWe-class nuclear reactors with redundancy and safety considerations (primary power source) / Multi-layer high efficiency PVs (secondary power source) / Automated dust removal system / Storage areas / Robotics for maintenance, EVA and unit operation / Virtual reality for leisure and psychological support (Tegmark [16]) / Expert system for medical, therapeutic, astrophysical and many other upcoming issues on board, its training and regulation (Bostrom [17], Kurzweil [18]) / The ethics of AI (Good [19]) / Redundant outdoor WiFi access point / Antenna / Easily accessible suits / Lifts traveling between three levels
Crew Special Activities	. Sleep . Leisure . Exercise . Sex . Personal hygiene . EVA . Mission dependent activities . Maintenance	Personal area suitable and quiet for sleep with flexible interior, controlled lighting, noise levels, ventilation, and temperature on second level / Privacy / Low gravity design criteria / Exercise area and recreational facilities / No pregnancy premise / Self-cleaning fabric / Minimizing laundry time and water usage / Minimizing EVA / Camp-out area / Providing access to spacesuits in airlocks as well as multiple storages inside unit
Psychological Aspects	. Claustrophobia . Solitude . Group dynamics . Visual perception / Light and color . Interactions with AI . Homesickness . Missing gravity . Fear of unknown . PTSD . Therapeutic needs	Large proportion of spaces / Interactive spaces / Meeting area for socializing and dinning / Architectural rhythm in the distinct spatial design / Defining regulatory instructions / Calming colors / Telecommunication facilities inside each unit / Reassuring protective measures in cases of unknown threats / Frequent therapy sessions with the local medical, psychological staff, as well as the Earth-bound support crew
Medical Conditions	. Medical care and surgery . Quarantine . Contamination . DCS . Deceased crewmember	Zonal separation / Installing medical and surgery packages in clean room conditions / AI assisted monitoring of the inhabitants / Medical waste disposal methods / Quarantine to avoid possible contaminations / Psychological support for grieving crewmembers
Emergency	. Emergency zonal separation . Unpredicted ET life form . Dust storms . Meteorite drops . Explosion . Fire	Automated sealed gates in order to separate damaged unit modules and contain the danger as far as it's possible / Quarantine area / Wind turbine (tertiary power source) / Wipers and shields for PVs / Artificial light supplies inside the unit / Vacuum fire prevention / Escape route to neighboring units
Construction Elements	. Materials . Foundation . Construction method . Structure . Sealing	Local excavations / Transportation from Earth / Using prefabricated 3D printed modules / Robotic assistance / Modular design, suitable for 3D printing using local materials / Sustainable configurations / Constant monitoring / Periodic maintenance / Redundant design

## Problem definition – mission specification

Having covered the general concerns applicable for a deep-space habitat design, precise definition of the mission specifications is the next required step. The current section presents a wide spectrum of probable/plausible mission specifications (Table 3), while highlighting the components that the mission for the current research is based upon.

**Table 3:** Mission specifications.

## Design process

The challenge of designing habitat units in an unconventional design context like Mars can be put into an architectural framework. Although the main concern is human survival, it is the responsibility of the architecture that goes far beyond the necessities of existence itself. In this approach, several ruling concerns and controlling conditions together with mission and site specifications define the required spaces and their organization, mainly for living and working, while there are qualitative features that flow in the design process from the functional programming to the presumed fixture assemblies. In other words, it is the architect's envision that establishes a new paradigm as a result of the interaction between insight and flow of the data.

In this light, the required spaces are first organized in terms of proximity and accessibility, followed by a fundamental layout with regard to space dimensions. This stage is similar to any other design scheme which is deduced by the kind of activities occurring in each space. Here, the main challenge is the psychological aspects of living isolated in a harsh environment like Mars that affects the sizing of the spaces. Referring to the Mars mockup projects in Antarctica, underwater habitats, or high mountains expeditions, the best number of crew members in terms of the level of deviance, conflict, and dysfunctionality problems is about nine. Hence, the proportion of spaces are calculated based on a module of 4 × 4 × 4 m³ growing in three steps, from four to six, and six to the maximum of nine residents in each unit.

What sets the MHUs apart is the unique way of composing spaces and form, as in every architectural design project is discussed under the flag of concept. In a rhombus-inscribed boundary, the devised interior gardens aligned with the main diagonal together with the peripheral (exterior) gardens provide each room with a view to the greenery. With the modules perpendicular to the interior gardens allocated to vertical access and services, the interspaces benefit from a dynamic horizontal circulation with distributary nodes. Another strategy that wraps the whole design appears in a hierarchical modular pattern that not only lets us penetrate to the spaces in a diverse yet well-organized manner, but also is used in the exterior shell pattern, not to mention its blessing in the construction process. Other than that, the adjustable exterior flow controlling mechanism known as Anti-Dust Settlement Membrane (ADSM) creates a paraboloid dome-shaped membrane on top of the rhombus boundary.

Finally, the design process would be incomplete without the consideration of construction methods. Enveloping details including the constituent layers are also presented, analogous to those architectural phase II detail designs. To make interactions possible for the AI-assisted 3D printing, known as the ultimate good solution for construction, geometric properties of the building envelope are defined by the equations. As all designs concerning human space mission, the currently proposed MHUs concept is under certain assumptions regarding the state of the technological advancements by the time of the missions. Even early-stage survival missions are to be performed under the assumption of availability of transportation to, and back from, the target planet/moon. As explained in Amini et al. [20], the MHUs are, however, designed for a much later point in human space exploration timeline, hence the deviation of the design from common shelters/pods proposed in the literature. This, nevertheless, does not essentially change the required technologies, but their capacity in quantity and volume, which will include more frequent transportation to the surface from the Earth and stations in orbit, better communication routes, reusable landing infrastructure, all of which are inevitable given the current state and slope of the research and development in governmental and private space-technology sectors. Regarding the construction of the MHUs, the proposed 3D-printing schemes using *in-situ* resources on the planet are, however, not futuristic at all, as there is a plethora of works in literature on the topic, one could refer to (Prater et al. [21]; Wekheiser et al. [22]; Schuldt et al. [23]; Meurisse et al. [24]; Truong [25]; Monsi et al. [26]). The available raw material on the Martian surface have been proven applicable in additive manufacturing of stand-alone walls and exterior facades, as well as being used as thick membranes burying the whole shelters to avoid radiation hazards.

Furthermore, a comprehensive top-down hierarchical coding system to address unit's components in its all levels is established which makes robotic construction viable. It should not be left unmentioned that the design process encompasses a kind of iterative methodology meaning to constantly check if the design output and its features meet the subject agenda or not.

## Martian Habitat Units (MHUs)

**Fig. 2:**
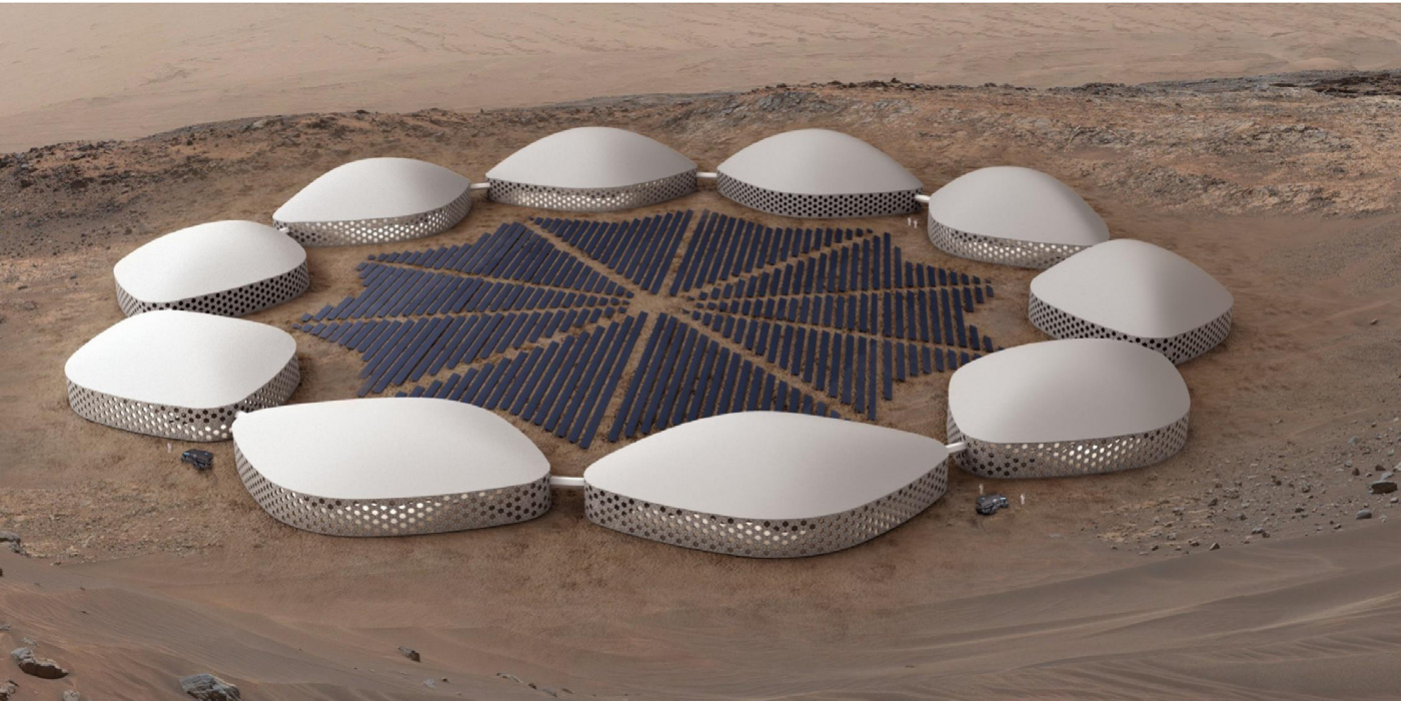
Cluster bird eye view.

**Fig. 3:**
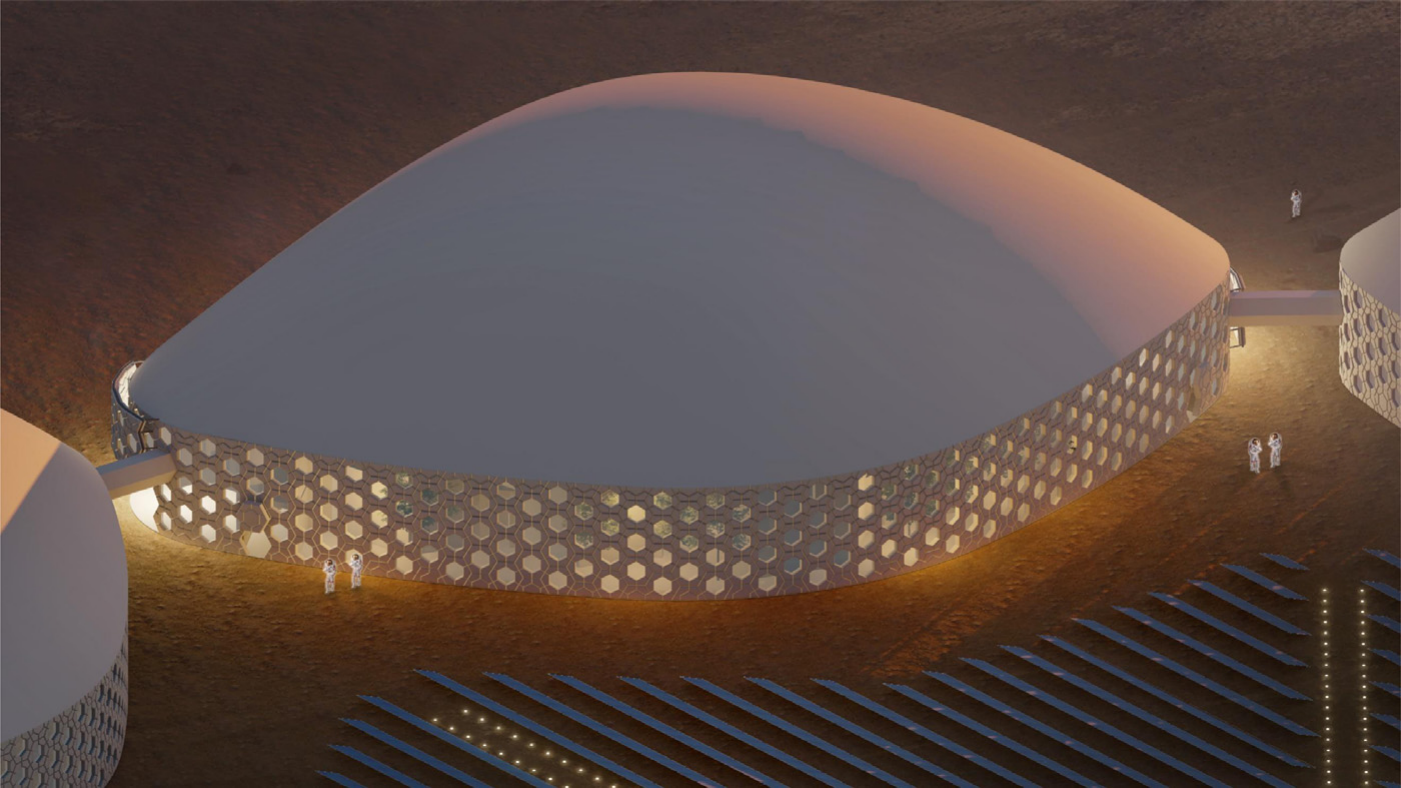
MHU bird eye view.

In response to the demands of a long duration research-based mission on the surface of Mars, the Martian Habitat Units (MHUs) are designed for specific needs of crew members during their years-long habitation on the hazardous environment of pre-terraforming Mars [20]. As an overall illustration of a cluster of MHUs, Fig. 2 represents a day-time bird-eye view, while a more focused view of one MHU is to be seen in Fig. 3.

**Fig. 4:**
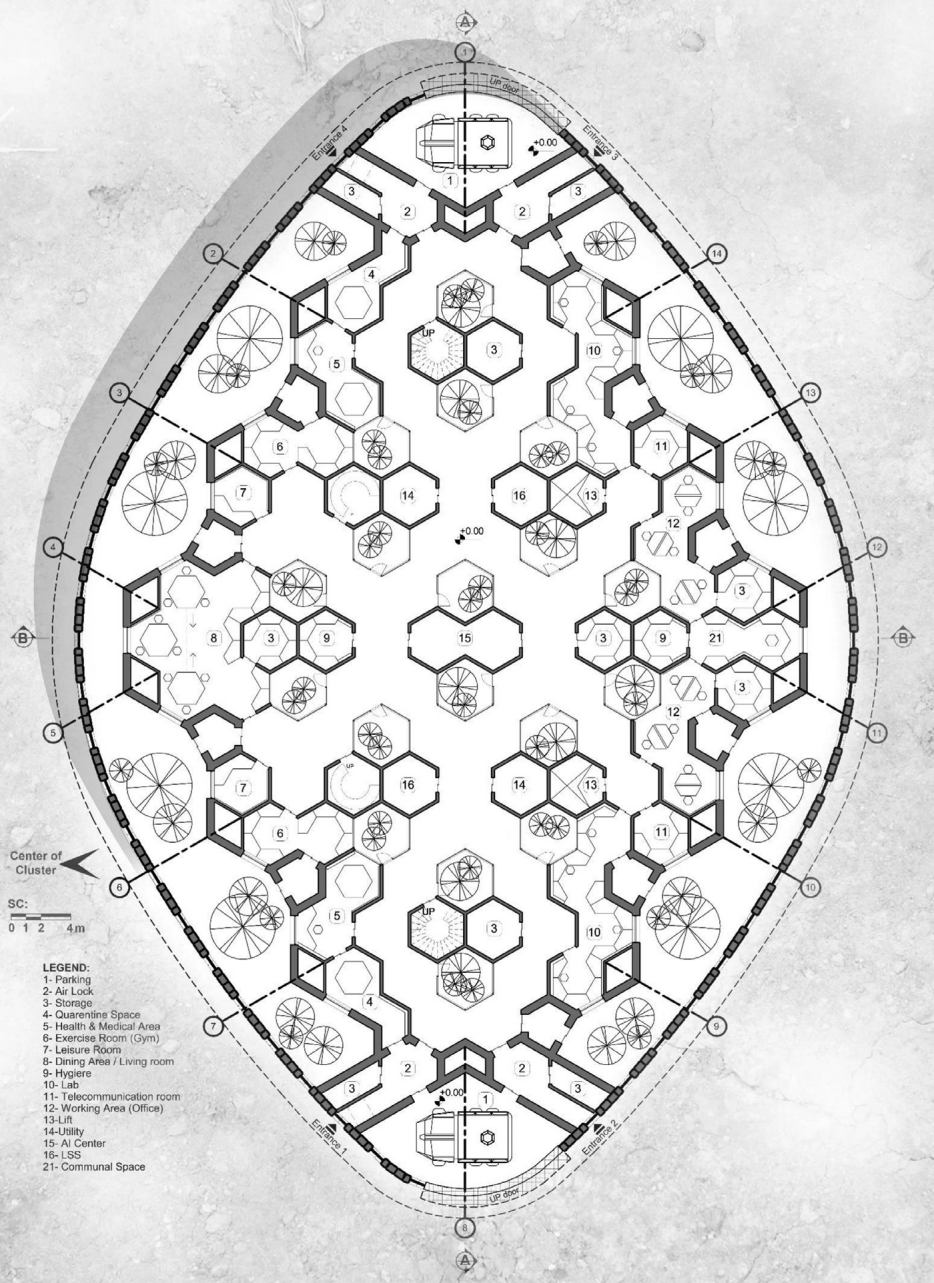
MHU Plan – Level I.

**Fig. 5:**
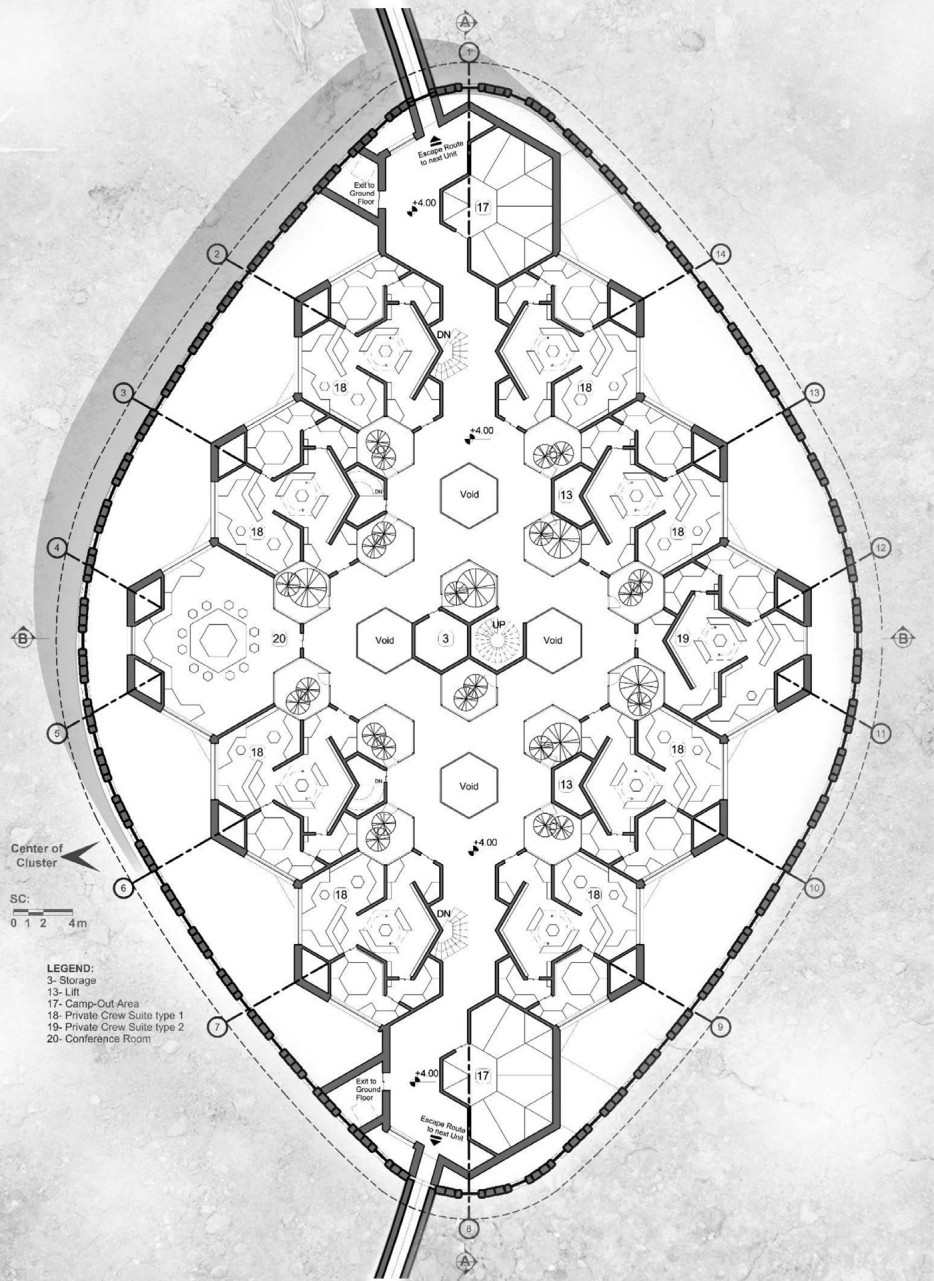
MHU Plan – Level II.

**Fig. 6:**
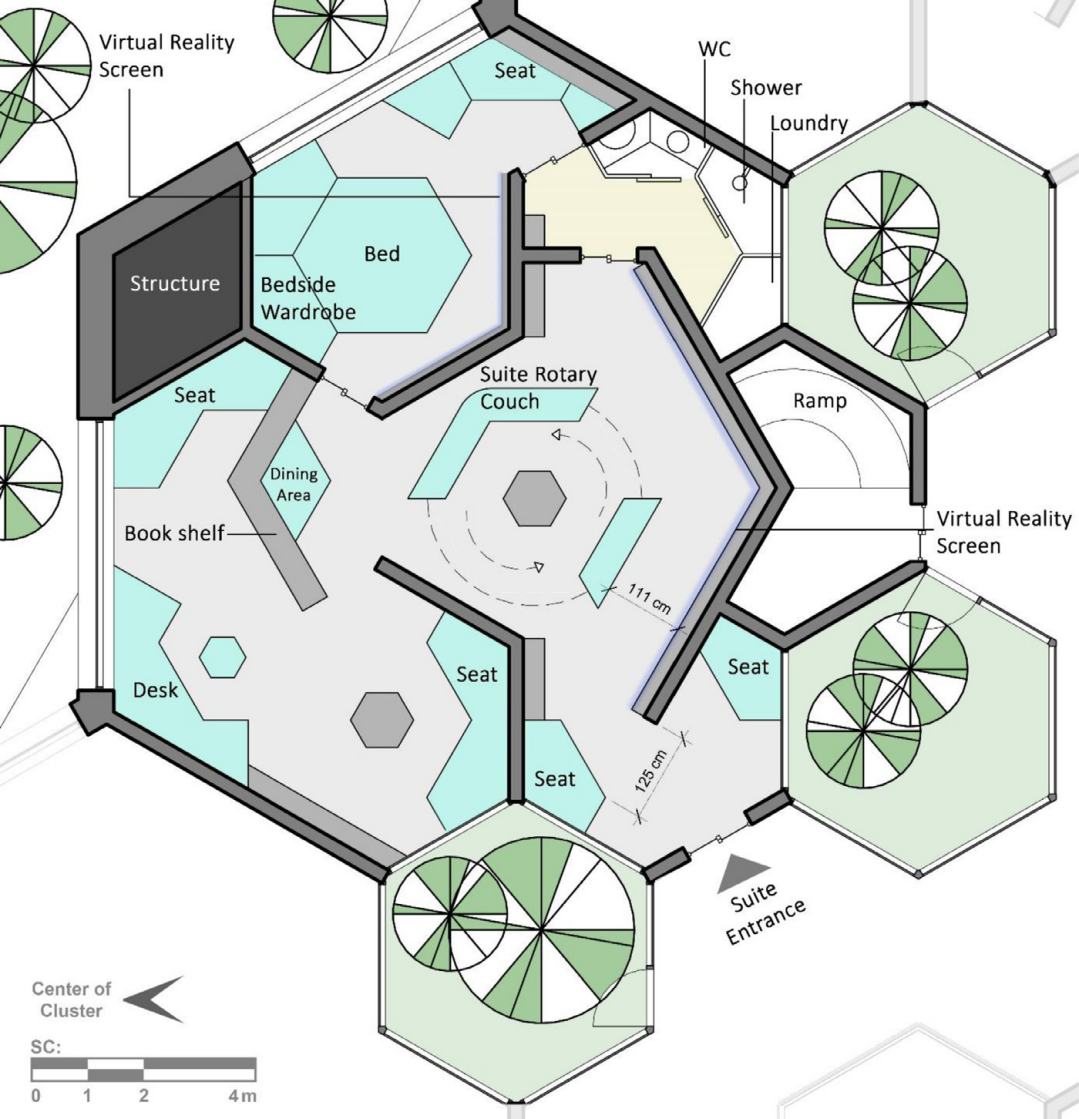
Crew suite plan – Type I.

In a bottom-up fashion, it should be noted that the building blocks of the design are hierarchical and interconnected hexagonal modules. A four-layered hierarchy of hexagonal construct with the growth rate of $\sqrt{3}$ among the layers is the main geometrical modularity to be witnessed throughout the design. The major hexagonal segments, with the side lengths of 6.495 m, is configured in a rhombus pattern of 4 by 4, forming the bulk of the MHU interior (Fig. 4, Fig. 5). A surrounding garden area engulfs the entire exterior of the plan, providing a more streamlined, curved exterior façade around the MHUs. All major functional sub-segments of the MHU are located on the circumferential regions of the interior plan to provide visual access to the enveloping garden and natural light during the daytime. In addition to that, there are multiple interior gardens in quest for a more comforting ambient. The MHU is designed as a two-decked habitat, with communal and working areas on the bottom level and crew member private suites on the second one (Fig. 6). A temporary shelter area is also placed on top of the second level and under the ADSM area, to be used in emergency cases.

Given the set mission and operational target of the MHUs, a research crew of 4–9 is planned to use each unit for accommodation over the span of 5 to 10 years, added by ∼500-day flights on both ends of the mission. This situation is extremely different from early-stage missions to Mars. Considering the radiation hazards, visual access to the exterior is reduced to the minimum [27]. And the harsh environment of the planet does not allow Extra Vehicular Activities (EVAs) of more than ∼6 h per week. There are seasons-long dust storms blocking all access to the exterior lasting for 100 to 150 days at each incident. There is limited communication to the Earth, with the delay of 20 min on each cycle, rendering any real-time conversation filled with 40 min pauses. Even with the availability of launchers, orbital dynamics of Mars and Earth do not allow travels outside of a short time window roughly every two Earth-years.

Confinement with the knowledge of unavailability of termination, fear of unknown, and extreme case of home-sickness governs the psychological well-being of the crew. Although such conditions are not testable in controlled research environment on Earth, there have been numerous attempts in replicating conditions analogous to that of such harsh missions. These have all concluded in opting strict protocols in providing personal/private spaces and limited daily demanded social interactions with co-inhabitants. The most common states are aggression, conspiracy-driven thoughts, suspicion, and overall feelings of being on edge. Therefore, monitoring and disconnection possibilities to handle harmful group dynamics should be in place.

Furthermore, the MHUs are designed with more emphasis on accommodating the researchers, which makes them distinct from the conditions of the current crew on the ISS. The very presence of the ISS crew in space is one of the over-arching topics of the research (i.e., human physiology in micro-gravity, etc.), and thereby conducted under the agreement of being constantly monitored; both physically and psychologically, while the researchers of the former, are to be habituated and settled whilst conducting research with pre-set numbers of work hours per day. Therefore, a higher level of privacy is in place, despite the similar necessity of constant monitoring.

In addition, the whole concept of the MHUs revolves around long-term life and settlement on Mars. The mission is defined distinctively from that of a survivalist crew with low recourses in early flights to Mars. The realization of the MHUs as a cluster of settlement habitats is possible after a certain number of early missions/flights to the surface, and going passed that phase, the nature of residence on Mars for the purpose of on-site research activities dictates higher quality of life. This colony is set as an intermediary step towards independent residence on Mars, where modified, location-oriented cultural thriving is also a goal, in opposed to mere survival [20].

Each MHU is designed with the maximum capacity of 9 crew members, and as there are 10 MHUs forming a circular cluster. The exterior envelope of each MHU is a cube of 73.96 × 51.7 × 14–24 m. And the height of MHUs are adjustable based on the wind conditions and dust-storm seasons. The interior of the cluster is carpeted with PVs and the Anti Dust-Settlement Membrane (ADSM) is implemented on top of the MHUs in form of an adjustable dome with a paraboloid surface function;

(1) $$\begin{array}{ccc} z & = & z\left(x,y\right)={z\left(x\right)|}_{\begin{array}{c} y=const.\\ \; \end{array}}=\left\{\left(\frac{H_{max}}{y_{max}^{2}}y^{2}-H_{max}\right)/{\left\{\begin{array}{c} \left(1-H\left[25.834\sin\frac{58.71}{2}\right]\right)\left(\sqrt{58.71^{2-y^{2}}}\right)\\ +\left(H\left[25.834\sin\frac{58.71}{2}\right]-H\left[28.852+8.13\cos\frac{89.05}{2}\right]\right)\\ \left(\sqrt{98.688^{2}-{\left(y+35.712\right)}^{2}}-63.501\right)\\ +\left(H\left[28.852+8.13\cos\frac{89.05}{2}\right]\right)\left(\sqrt{8.13^{2}-{\left(y-28.852\right)}^{2}}\right) \end{array}\right\}}^{2}\right\}x^{2}\\ & & \;\;-\;\frac{H_{max}}{y_{max}^{2}}y^{2}+H_{max} \end{array}$$

where, *H* is the Heaviside unit step function. The maximum height of the ADSM is obtained based on the wind flow field in the local areas near the cluster, and its main function is to prevent or minimize dust settlement on the PVs located in the interior of the cluster, in dust storm seasons. The height alterations are maintained through hydraulic actuators on the central regions of the MHU and are aided with pressurizing the entire ADSM volume with compressors feeding the Martian atmospheric content.

Here is a brief explanation of the major features of MHUs;

*Extreme Redundancies* - As a requirement to ensure safety and reliability for both systems and humans, a three-fold redundancy in an “Active/Active” operational mode is considered. This fail-safe feature impacts the whole design of the MHU from spatial sizing to mechanical and electrical properties in life support systems.

*Flow Controlling Measures* - To preserve solar panels, from seasonal sand-storms, an inflatable elastic membrane, which is called Anti Dust-Settlement Membrane (ADSM), has been designed on the top of each unit. It deviates the wind path by being elevated during the storm seasons.

*Double-Layered Pressure Vessel* - Given the pressure difference between Martian atmosphere and the residents’ units, we came to a strategy other than the rigidity of structure and façade, i.e. an interspace where the pressure is maintained at around 80% of that of the 1 atmospheric condition. Hence, the pressure increases from outside to inside in two steps.

*Interior Gardens’ Lighting System* - As there is no way to access direct sunlight (in case of availability) in the interior gardens, known as the visual segments in our design, the presumably devised light-tubes make the concept of natural lighting possible.

*Fenestration - Virtual Reality Interfaces* - The importance of daylight and windows in architecture as well as its relationship with mental health has been proven. Due to the unstable conditions on Mars, it is not possible to use windows such as that of the Earth for the units. In order to feel connected to the outside, virtual reality LCDs, which represent the external environment without being really connected to it, are installed on the walls.

*Enveloping Garden* - Apart from abating the pressure difference, the second-layer of the vessel is home to the peripheral garden which functions as the main source of food supply in a closed loop physio-chemical life support system. It also allows the atmospheric revitalization by ejecting proportions of carbon dioxide from inside into the garden, which is also a necessity for planting.

*Solar / Wind Power Sources Interactions* - Solar, wind, and nuclear power interact together to supply energy in the MHU cluster. Solar and wind power act as backups for nuclear power when it is not available as a primary power source; Thus, solar power is considered as the secondary energy source, and wind power will support energy demand in dusty air condition when the energy generation by solar panels decreases.

*Additive Manufacturing* - AI-based 3D printing with the use of *in-situ* material is the ultimate solution for the construction due to the heavily technology-oriented field of Mars design, and to minimize human costs and efforts in the harsh environment of Mars.

*Modular Design* - The modularity as the core concept of the whole design is not only a value but rather a necessity in different stages. For this, a 4-layered hexagonal pattern of modules allows the organization of spaces, and the hierarchy of modules, also represented in their sizing, makes the geometric diversity possible.

*AI-Assisted Construction* - According to the mission specifications, the crew members are to live a thriving life for long term durations. Based on the mentioned approach, robotics will construct MHUs before the settlers land on Mars which is possible by the AI assistance and the developed of the components’ addressing coding system.

*Extendable to Higher Capacities* – To accommodate different number of crew members according to the mission, an adaptable capacity is adopted. In the flexible design of units, known as extendable capacity, the crew numbers will extend 50% in three steps where the minimum capacity is 4.

*Escape Routes* - On the second level of each unit, there are two bridges, named escape routes, for emergency cases. Escape routes are the final choice to save the crew by connecting units to the neighboring ones.

*Emergency Zonal Separation Gates* - To improve safety in emergency cases, the MHU should be able to resist, manage and mitigate the situation. Hence, the MHU is designed as a symmetrical building to maintain its functionality in emergency cases even if some part of the unit is divided by zonal separation gates.

*Shelter / Bunker* - The 3rd level of the MHU is dedicated to a temporary shelter assuming an emergency and when no other evacuation plan is viable. Food, medical kits, communication devices, and beds are stored in this place.

## Power generation methods and concerns

MHU as a self-regulating closed system depends highly on multiple co-functioning subsystems. The input data from *in-situ* sensors and monitoring satellites facilitate the logical decision-making to act when necessary, either automatically by intelligent controllers or manually by the crew. To ensure the continuous operation of these subsystems, most notably the life support systems, a redundant and robust design of power generation sources is essential. Considering the long seasons and challenging atmospheric conditions of Mars, it is evident that to ensure the feasibility and safety of the long-duration missions for the inhabitants, nuclear fission reactors should be considered as the primary power generation source. By the time of this writing, NASA's Kilopower reactors are considered. Their compact design and self-regulating scalable fission power allow a redundant configuration of multiple 10kWe-class reactors to be used to supply the consumable energy. Also, their reliability and modularity are in line with the modular design concept behind the MHUs. As the secondary power generation source, a solar farm has been designed to provide power for 10 MHUs in the middle of the cluster. As the performance of even highly efficient photovoltaic cells depends on the sun angle and the cleanness of the cells, it is predictable that there will be a drop in power during dark dust storm seasons on Mars. In an innovative approach, wind turbines have been considered the tertiary power generation source to use the available storm energy as well. To reduce the dust settlement on PV cells, the ADSM height of every MHU is adjustable according to the wind velocity and amount of dust on cells. Apart from that, the robotic arms and telepresence technologies will help maintain the cells and remove the inevitable residual dust while reducing or eliminating the EVA. The generated power is regulated and distributed among all the operating systems. With continuous communication with AI monitoring systems, the power control and regulation unit also determines which combination of these methods should be working simultaneously and to what extent at a time. The excess power is stored in Li-ion batteries for emergency cases although with the current configuration of power generation technologies the need to store power is minimal. Radioisotope Thermoelectric Generators (RTG) are also considered to be used in short and rare situations to facilitate remote or crewed expedition vehicles or instruments and when emergency electrical power is needed immediately. The reason for this decision is the independence of RTG from the main power sources and their easy to shield against α-radiation emission. But regarding RTG lifespan and the operating nuclear reactors, safe containment of the radioisotopes, shielding and the possibility of nuclear contamination are of concern and require concrete planning.

NASA's Human Spaceflight Architecture team has assumed four 10kWe Kilopower reactors for the 40-kWe first human Mars surface mission requirement that supplies a crew of 4–6 astronauts. With a conservative assumption of 30 kWe per crewmember per day, we consider a minimum of 18 and maximum of 27 Kilopower reactors per MHU based on an evaluation of the daily energy consumption for the crew with a high safety factor. It is estimated that nine crew members will demand a 1087.61 kWh/day total energy. With a safety factor of 3, this amount is 3262.8 kWh/day. The following tables (Table 4, Table 5, Table 6, Table 7) summarize the technical details and assumption considered to meet the total energy demand per MHU. PV cell area is calculated based on the worst-case scenario when the solar flux is the minimum. The wind turbine is designed according to its possible peak performance during a dust storm as the power produced by the PVs is under the nominal value.

**Table 4:** Primary power generation source nuclear fission reactor kilopower.

Items	Specifications
Fuel	Solid U235
Cooling	Passive sodium liquid heat pipes
Power Converter Configuration	Stirling
Control mechanism	Boron carbide control rod
Operation period	All Martian year
Reactor thermal power	43.3 kWt
Power conversion output	10 kWe
Converter efficiency	∼30%
System efficiency	∼25%
Number of reactors per MHU	18 - 27
Daily operation hours	24 h and 40 min (average sol)
Total daily energy	4438.8 – 6658.2 kWh/day
Safety factor	1.5
Consumable daily energy per MHU	2959.2 – 4438.8 kWh/day

**Table 5:** Secondary power generation source multi-junction photovoltaic cell.

Design Parameters	Specifications
Area per MHU	3400 m^2^
Efficiency	33%
Operation period	All Martian year
Lowest daily average radiation on tilted surface	0.644 kW/m^2^ (Ls = 75)
Performance ratio	75%
Daily operation hours	9 h
Average total energy	4877.3 kWh/day
Safety factor	1.5
Consumable daily energy per MHU	3251.53 kWh/day

**Table 6:** Tertiary power generation source horizontal axis wind turbine (HAWT).

Design Parameters	Specifications
Diameter	40 m
Hub height	50 m
Operation period	Dust storm season
U* (40 m) during peak performance	35 m/s
Power coefficient	59.3%
Daily power generated	776.4365 kW
Daily operation hours	8 h
Number of towers	5
Total consumable daily energy	31,057.46 kWh/day
Consumable daily energy per MHU	3105.74 kWh/day

**Table 7:** MHU energy consumption.

Items	Values
MHU total energy consumption (9-crew)	1087.61 kWh/day
Safety factor	3
Total daily energy demand per MHU	3262.8 kWh/day

## Manufacturing process

Due to extreme environmental conditions and to reduce the crew efforts in manufacturing processes, the habitats are built prior to the crew arrival and via programmed robotic agents. Considering the modular design of the hexagonal-shaped prime module, interplanetary transportation costs, and the maintenance of the units, the additive manufacturing method and 3D printing the main structures and modules using *in-situ* materials, is the only optimal and robust solution for the construction of the MHUs.

This process starts with robotic arms providing the granulated Martian rigorous as the raw material for the 3D printers. The printed architectural elements include the side length of four hexagonal shapes with an internal ratio of factor $\sqrt{3}\;$between every two consecutive layers. The various layout and interaction of these four layers of the prime hexagonal module are used to assemble the unit and define the interior spaces of MHUs. To ensure the correct assembly of the modules and later address all the unit's components, as some might need maintenance, repair, or replacement, a coding system is introduced based on the geometrical features of the prime module. These codes are the raw data input to the Al-aided construction process, as the size, purpose and position of each element are also dictated.

As the exterior and the main interior spaces of the MHU are constructed, the unit provides the initial protection for crew arrival. It is assumed that the previously sent Kilopower reactors for manufacturing purpose provide enough power for the manufacturing process explained and the installation of the Life Support Systems (LSS), including their remote initial tests. The remaining components that are also modular in the design must be transported and assembled when the crew moves in. These include the unit components that cannot be produced using *in-situ* materials, electronics, spare parts, instrumentation, and equipment needed for cultivating plants, maintaining unit and vehicles, etc. After the crew has successfully put the LSS in full, redundant operation, they can settle in. Private spaces and furniture are also 3D printed simultaneously and are compatible with the number of inhabitants and each crew member's values and needs. Instalment of secondary and tertiary power sources are later done under the supervision of the crew.

## Future works – full scope of the project

Having a methodical approach towards the big picture of the human missions on Mars, the MHU project has been designed with the starting point of space technological and architectural conceptual and preliminary design (and in some areas up to the detailed design) of the MHUs (Amini et al. [20]). The current manuscript presents the methodological aspects of the design process, which then together have bifurcated to three main research areas; namely the Life Support System (LSS) assessment and trade off study, natural lighting simulations and evaluations for the Martian surface, and numerical study of the exterior flow field as a proof-of-concept level investigation for the ADSM, as well as obtaining the wind loadings exerted on the body and the distribution of convective heat transfer coefficient on the facades. The latter CFD study should also be verified hand in hand with wind tunnel measurements for a single MHU. By the time of issuance of the current manuscript, the mentioned block is being under progress, set to be communicated as separate publications in near future.

The data obtained through this block on the consumption and specifications of the final chosen LSS, the required level of artificial lighting to compensate the dimmer surface of Mars in comparison to human crew requirements, and the convective heat transfer capacity of the exterior flow field, will then lead to a comprehensive study on energy balance and thermal performance of a single MHU. This block will then result in economic studies for the manufacturing and installation cost analysis of a cluster of MHUs. In parallel, each of the three branches of the previous block delve further into the corresponding detailed studies such as LSS and AI combination using control theory and machine learning schemes, 3D CFD and parallel wind tunnel testing on the full cluster of MHUs, and structural design of the 3D printed units.

**Fig. 7:**
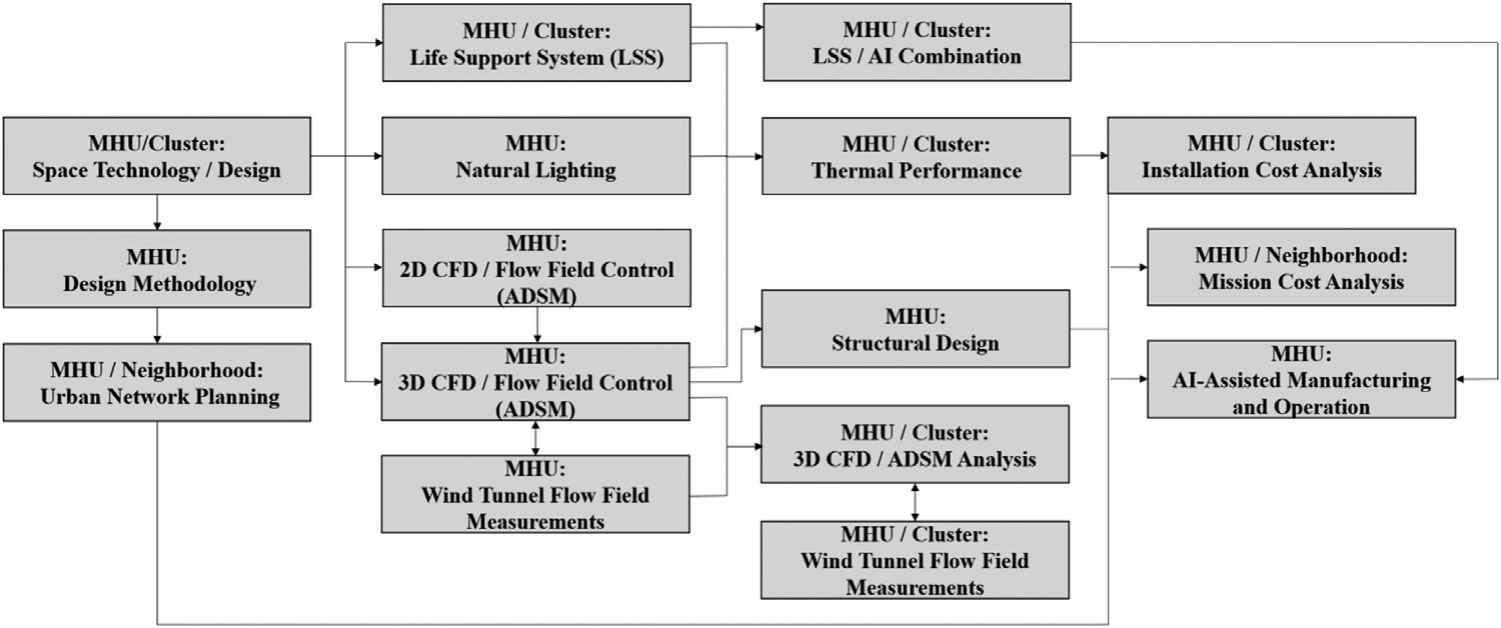
Project bifurcation block diagram.

Finally, a separate branch of the project on urban design and planning of a network of MHUs/Clusters on a neighborhood level. This branch could be integrated with the final results of cost study resulting in a neighborhood level economic investigation and mixed with the LSS/AI combination leads to a comprehensive AI-assisted manufacturing and operation of the units / full mission. Fig. 7 sums up the interactions and order of the mentioned blocks in a flow chart.

## Conclusion

The Martian Habitat Units (MHUs) are designed to operate as a research base for long duration presence of scientific crew for in-site exploratory missions. The time era corresponding to the manufacturing of MHUs coincides with pre-terraforming phase on Mars, however, presumes availability of flights to and back from Mars, and presence of orbiting space stations around the planet. The mission definition with 9 crew members on each MHU among the 10 in each cluster is then in compliance with the accessibility of *in-situ* resources and feasibility of years-long human crew research missions on Mars.

In response to sever challenges on the harsh environment of Mars, higher than conventionally dictated levels of safety measures and redundancy schemes for all static and dynamic procedures in the design. Energy sources are in harmony throughout the year, as dust seasons reduce the capacity of solar power, while increasing the wind flow velocities beneficial for wind turbines. Simultaneously, flow controlling and streamlining mechanisms protect the interior of the cluster, where the PVs are installed, from dust settlement. This is while the active energy sources, i.e. nuclear reactors, are always in circuit and only reliant on wind and solar sources as backups.

AI-assisted life cycle starts for MHUs from the first processes done by robotic components, while constructing the major bulks of the MHUs from *in-situ* materials, before the arrival of human crew members. This strong presence of AI is then carried on during the operation of the MHUs, and their maintenance.

Considering the 3D printing construction method and utilizing *in-situ* resources to build MHUs, reduce the amount of construction material to be transported significantly. On one hand, it can be assumed that the required payload would be consisting of instrumentation, robots, and large-scale printers instead of raw materials. On the other hand, the assumed timeline has been specifically one of the design drivers of MHUs for a scenario in that human comfort and socialization as part of a research expedition are prioritized while ensuring human survivability.

We have aimed to isolate the mentioned scenario to design a habitat with a set of requirements and concerns explained in Table 2 of the manuscript. Furthermore, the modularity of this design concept is chosen to account for the identified and unidentified uncertainties about a mission that would take place decades later. In this regard, each unit is designed to operate independently of surrounding units, and the interior of the unit follows repetitive patterns and extreme redundancies, which can be easily adapted to the needs of the crew at the time. Hence, realizing such a mission, i.e., construction of at least one safe MHU with an interior design suiting the minimum number of expected crewmembers, would not require a large number of launches in the first place.

One should also acknowledge however, it is difficult to estimate the exact number of launches needed, but considering the current launcher capacity and its rapid advancements, for instance, the Falcon 9 rocket lifted 56 Starlink satellites, with a total weight of 17.4 tons, into low Earth orbit and the Starship is designed to have a payload capacity of 150 tons to low Earth orbit in a fully reusable configuration and 250 tons when expended, it can be concluded that such a futuristic mission would not be unrealistic at all.

Considering all the above-mentioned concerns and criteria, and numerous others outside the scope of the current manuscript, in a functional compliance with the environmental challenges of living on Mars, with the eventual target of maintaining a thriving life-style more than a mere shelter has led to the design of MHUs and their surrounding cluster. The current paper focuses on the design methodological aspects and steps of the project.

## Declaration of Competing Interest

The authors declare that they have no known competing financial interests or personal relationships that could have appeared to influence the work reported in this paper.

## Data Availability

No data was used for the research described in the article.
